# Family physician enabling attitudes: a qualitative study of patient perceptions

**DOI:** 10.1186/1471-2296-14-8

**Published:** 2013-01-10

**Authors:** Catherine Hudon, Denise St-Cyr Tribble, Gina Bravo, William Hogg, Mireille Lambert, Marie-Eve Poitras

**Affiliations:** 1 Département de médecine de famille et de médecine d’urgence, Université de Sherbrooke, Québec, Canada; 2 Centre de santé et de services sociaux de Chicoutimi, Québec, Canada; 3 École des sciences infirmières, Université de Sherbrooke, Québec, Canada; 4 Département des sciences de la santé communautaire, Université de Sherbrooke, Québec, Canada; 5 Department of Family Medicine, University of Ottawa, Ottawa, Canada

**Keywords:** Power (psychology), Enablement, Patient-centred care, Family practice, Primary health care, Chronic disease

## Abstract

**Background:**

Family physicians frequently interact with people affected by chronic diseases, placing them in a privileged position to enable patients to gain control over and improve their health. Soliciting patients’ perceptions about how their family physician can help them in this process is an essential step to promoting enabling attitudes among these health professionals. In this study, we aimed to identify family physician enabling attitudes and behaviours from the perspective of patients with chronic diseases.

**Methods:**

We conducted a descriptive qualitative study with 30 patients, 35 to 75 years of age presenting at least one common chronic disease, recruited in primary care clinics in two regions of Quebec, Canada. Data were collected through in-depth interviews and were analyzed using thematic analysis.

**Results:**

Family physician involvement in a partnership was perceived by participants as the main attribute of enablement. Promoting patient interests in the health care system was also important. Participants considered that having their situation taken into account maximized the impact of their physician’s interventions and allowed the legitimization of their feelings. They found their family physician to be in a good position to acknowledge and promote their expertise, and to help them maintain hope.

**Conclusions:**

From the patient’s perspective, their partnership with their family physician is the most important aspect of enablement.

## Background

The family physician frequently interacts with people affected by chronic diseases [1,2]. He or she is in a privileged position to enable people [3], by his attitudes and behaviours, to increase their individual empowerment [4-6]. The enablement process is defined as a professional intervention aiming to recognize, support and emphasize the patient’s capacity to exert control over his or her health and life [4]. Individual empowerment translates into a growing awareness of one strengths, improved self-esteem, decreased anxiety or sadness, improved decision-making, development of new skills and moving towards action [7,8].

Soliciting patient perceptions about how their family physician can help them become more empowered is an essential step towards promoting enabling attitudes among these health professionals. We found no evidence in the literature of papers addressing patient perceptions of physicians enabling attitudes. Thus we looked for research that described patient perceptions of different aspects of patient-centred care. Patients valued the close trust-based relationship they had with their family physician [9]. They confided that consultation style, continuity of care and a tailored or individualized approach may have an important impact on their self-confidence and on their ability to cope with the strains of illness [10,11]. Patients relied on their family physician to clarify information and treatment options provided by the hospital [9]. They also expressed a need to see their struggle acknowledged and their illness experience legitimized [12,13].

The results of these studies contribute to a better understanding of the doctor-patient relationship, but so far none of them have focussed on processes of enablement to promote patient empowerment. In the present study, we aimed to identify family physicians’ enabling attitudes and behaviours from the perspective of patients with chronic diseases.

## Methods

### Design

Qualitative description was used as our research approach as defined by Sandelowski [14,15]. This allowed us to provide a comprehensive description of enablement in plain language while staying close to the data, minimizing researcher influence on the data and reflecting patient perspectives [15].

### Participants

The study was conducted in the Saguenay and Sherbrooke regions of the Province of Quebec (Canada) between December 2009 and December 2010. Each region’s population is approximately 150,000. A convenience sample of 12 family physicians was selected by researchers according to their gender and experience to reflect different practice styles, typical of family practitioners in Canada. At first, they were instructed to choose French-speaking patients based on the sampling criteria provided. More precise instructions were later given for the last participants recruited regarding age, gender and chronic disease to represent younger and older people of both genders with different chronic conditions (maximum variation) [16]. Participants had to be between 35 and 75 years of age and affected by at least one of the chronic diseases most frequently seen in primary care: osteoarthritis /arthritis or other significant musculoskeletal condition, hypertension, hyperlipidemia, diabetes, heart disease, chronic obstructive pulmonary disease/asthma, depression/anxiety [17,18]. Patients also had to be willing to share their opinions about the research topic. Patients with cognitive impairment, uncontrolled psychiatric illness, or a serious hearing deficit, as assessed by their family physician, were excluded from the study.

### Data collection

In-depth individual interviews were used to capture the richness and nuance of experiences with a focus on participant perspectives [19,20]. After providing written informed consent, each participant completed a short sociodemographic questionnaire and participated in a one-hour interview conducted by an anthropologist trained in qualitative research (ML). The interview guide (Additional file 1) included open-ended questions asking patients to describe: 1) their health status and its impacts on their life; 2) their encounters with their family physician; and 3) their family physician’s role in helping them increase control over and improve their own health. The last item aimed to elicit what makes patients become more aware of their strengths, develop self-esteem, decrease negative feelings (anxiety or sadness), make decisions, develop skills or take action [8]. The interviewer adapted her language and way of asking questions to the education level of participants. The interview guide was developed to identify positive attitudes and behaviours promoting patient empowerment. Negative experiences were also sought to highlight enablement attitudes or behaviours that were lacking. The interview guide was pre-tested with four patients (not included in the study) and revised to enhance comprehension. All interviews were audio-taped.

### Analysis

As our interpretation is influenced by our knowledge and previous experience [21,22], we decided to be clearly explicit about the work previously conducted on a similar subject. We used the six main themes of our literature review on patient-centred care in the context of chronic disease management in family medicine [23] as a conceptual framework. However, this framework was used only at the analysis stage and not to develop the interview guide in order to preclude any bias.

Two authors from different professional backgrounds (CH, an experienced general practitioner and ML, an anthropologist) read the transcripts and analyzed them independently using mixed coding as described by Miles and Huberman [24]. The relevant features addressed in the interviews were organized into a grid according to codes based on the conceptual framework as well as new ones emerging from our analysis. Initial codes of the conceptual framework could be removed if not expressed by the participants. Through thematic analysis, codes were refined and transformed into themes, and further divided into sub-themes [25]. Final themes were voluntarily worded positively. Since negative instances of a concept can shed further light on this concept, negative experiences were also sought. So the participants worded certain experiences negatively. In these circumstances, we tried to come to an understanding with the participants of what their physician could have done better to help them become more empowered. The choice was made, in our results to keep verbatim that demonstrated positive behaviors but negative verbatim were used in the analysis as well.

Discrepancies and disagreements were discussed with other co-researchers (MEP, DST, GB). Pair debriefing, triangulation and team validation minimized the influence of researcher subjectivity, thus improving the credibility of the work [26]. Transparency in analysis and reporting was achieved by providing extensive verbatim quotes. Interviews were conducted until the point of data saturation was reached [20,26]. Our sample size (30 interviews) is in line with recommendations in the literature on descriptive qualitative studies [14]. NVivo 2.0 software (QSR International Pty Ltd) was used to manage the qualitative data.

### Ethical considerations

The study was approved by the research ethics boards of hospital centres in both regions: *Centre de santé et de services sociaux de Chicoutimi* and the *Centre de recherche Étienne-LeBel du Centre hospitalier universitaire de Sherbrooke* (2009–014). This study was based on the usual ethical principles, such as each person’s right to refuse to participate in the study and to withdraw at any time, as well as respect for all participants and protection of their privacy. Each person recruited received all the information necessary to provide free and informed consent.

## Results

### Participants

Thirty patients, 17 women and 13 men, were interviewed. Table 1 shows the sociodemographic characteristics of the sample. Additional file 2 provides more detail on each participant.

**Table 1:** Sociodemographic characteristics of the sample

**Characteristic**	**Number**	**Percentage (%)**
**GENDER**		
Male	13	43
Female	17	57
**PLACE OF BIRTH**		
Quebec	28	93
Other province of Canada	2	7
**EDUCATION**		
Grades 1-7	1	3
Grades 8-12	11	37
Postsecondary studies or college	8	27
University	10	33
**ANNUAL FAMILY INCOME (CAD$)**		
< 10,000	2	7
10,000 to 19,999	6	20
20,000 to 29,999	2	7
30,000 to 39,999	2	6
40,000 to 49,999	6	20
50,000 +	12	40
**MARITAL STATUS**		
Married / Living with partner	19	63
Separated / divorced	5	17
Widowed	2	7
Single	4	13
**CHRONIC DISEASE**		
Depression/anxiety antecedents	14	47
Arthritis/osteoarthritis/other joint problems	12	40
Diabetes	11	37
Emphysema/chronic bronchitis/asthma	5	17
Hyperlipidemia	19	63
Hypertension	16	53
Cardiac or vascular disease	15	50
**MEAN AGE (range)**	60.5 (35 to 75)	

### Qualitative results

This section presents the attitudes and behaviours of the family physicians that enabled participants. Table 2 identifies these results in terms of themes and sub-themes. Verbatim quotes are identified by interview participant number.

1. Developing a partnership

Participants reported that developing a partnership with their family physician over time is a key element to promoting their empowerment. Twenty-eight participants addressed partnership in regard to a trust-based relationship and 23 participants in terms of decisions and choices to be made.

A. A relationship based on trust

By helping the patient feel comfortable, showing empathy and respect, being sincere, demonstrating professionalism and engagement, spending enough time and fostering relational continuity, the family physician contributed to the development of a relationship built on trust. Most participants stressed the importance of this alliance in promoting their empowerment. For some, it played a key role at some point in their life. Five people spontaneously compared their physician to a family member or a friend.

**Table 2:** Themes and sub-themes emerging from the thematic analysis

**Themes**	**Sub-themes**	Verbatim*
1. A) Developing a partnership: a relationship of trust Table 2	Helping the patient feel comfortable	I feel very good with Doctor X, I am not embarrassed to tell him everything. (22)
	Showing empathy	She is so always ready to listen to the person, that if I have problems, I know I will be able to talk to her. (14)
	Showing respect	You don’t feel things are imposed on you but you don’t feel judged either. (16)
	Being sincere	She is good, she tells you the truth, and she doesn’t hide anything, you know. (21)
	Demonstrating professionalism	Me, her relationships with one and another don’t interest me. (10)
	Demonstrating engagement	When my husband was in his last weeks, she was pregnant, she was about to stop working. She would go see him once or twice a week. That really touched me. She did not have to do that. (30)
	Spending adequate time	He does not look at the time, he listens, looks at you and he catches everything you say. After that, he responds to what you asked him. (2)
	Fostering relationship continuity	I think that by seeing each other, we developed a privileged contact. (17)
1. B) Developing a partnership: Finding common ground	Informing	He says just enough. He explains what you want to know. (2)
	Providing results	She comes and reads my results she just received. I am encouraged when I do something good. (13)
	Taking preferences into account and respecting choices	He accepts your choices. You are the one who decides. (16)
2. Promoting patient interests in the healthcare system	Demonstrating professional competence	I would expect him to use all his medical knowledge to find the problem. (20)
	Fostering collaborations with other health professionals, specialists, community resources and alternative and complementary medicines	It is even her (physician) who gave me the name of an acupuncturist that I went to see. She oriented me. (16)
	Fostering continuity of care	She knew everything the cardiologist was doing with me, because he would convey the information to my family physician. (24)
	Fostering accessibility to care	So that, knowing I can call him… that is less stressful. (3)
	Accompanying in the steps to be taken	He helped me a lot step by step to get to the surgery. (1)
	Ensuring patient safety	He left a message on my answering machine. He wanted to know how I was doing. He thought… I looked so bad that he was worried. (19)
3. Starting from the patient situation	Knowing the antecedents and the health status of the patient	She knows me from A to Z. (24)
	Knowing the feelings (anger, sadness…)	When I found out I had diabetes. He noticed that I was shaken … (19)
	Knowing the repercussions	She will ask me: what about the pain, how are you doing? How are your days? (9)
	Knowing the expectations	And in the end, he will ask, do you want anything? (18)
	Knowing the personality	He knows I am fearful. He went to get a book with an image to show me where it was in my knee and how he would give me the infiltration… I thought that was kind of him. (2)
	Knowing the family context	She always asks about my grandchildren. You know, there is something there … (14)
	Knowing about the work status	In regards to work…you know we cover that. (16)
	Knowing about leisure time or activities	My leisure activities, if I practice sports. She asks about everything. (14)
	Knowing about the life context	She asked me what kind of summer I had… you see that she is interested in your life… that’s a lot. (25)
	Addressing the subject of sex	I have a follow-up on everything… even on the issue of sex. (18)
4. Legitimizing the illness experience	Recognizing the suffering	God you suffer, it makes no sense… you know, she can’t believe how I am so organised. (9)
	Managing emotions linked to the absence of a diagnosis or an uncertain or worrisome diagnosis	They want to help me on that aspect… because for me, not to know what it is, it’s difficult. (8)
5. Acknowledging and promoting the patient’s expertise	Promoting healthy lifestyle habits	He also mentions things to do or to not do… that are not related to medication. Therefore, not everything is settled by a pill…There are other things all around that we look at. (5)
	Encouraging self-care	I have a prescription but I am the one who manages it. (16)
	Advising	The advice she’s going to give me, for sure I will take it cause I know it will work. (24)
	Fostering greater awareness	He always had the right way to make me understand things that I really did not want to understand. (22)
	Fostering self-confidence	He knows I can understand… occasionally, he says: “Now, you know what to do, it’s up to you, it’s your responsibility, go ahead”. (5)
6. Helping the patient maintain hope	Playing it down	He can help me put things in perspective. (15)
	Supporting	If I have concerns, Dr X reassures me. That allows me to be free. (2)

*Verbatims were translated from French to English for the purpose of this paper.

*“I do trust my family physician a lot. When I was depressed, if it wasn’t for her, I would probably not be here today to chat with you”*[23]*.*

*“By getting so used to her… she was almost like my sister”*[23]*.*

B. Finding common ground

Participants also described a partnership in regard to decision-making and choices. They expressed a need for reliable information, to be informed of their test results, and respect for their choices.

*“Together, we look at the positives, the negatives of each thing… But I am the one making the decision”*[15]*.*

2. Promoting patient interests in the healthcare system

The issue of difficulties in accessing a saturated care system was often raised as it can increase or generate significant anxiety and hamper patient self-management. Twenty-eight participants stressed the need to have their physician involved in promoting their interests and their safety in the health care system, in particular, regarding accessibility, continuity and coordination of care. Some also expressed the desire for orientation on matters of alternative and complementary medicines and community organizations.

*“I think that it’s the family physician the orchestra conductor, who is the best person to see that the patient is well supported”*[16]*.*

3. Knowing and starting from the patient’s personal situation

Eighteen participants reported that their physician maximized the impacts of his or her interventions by becoming aware of and taking into account their personal situation. Understanding how the person perceives and experiences his or her different health problems and knowing the environment and distinctive contexts in which he or she evolves, “*puts the physician in a very good position to propose a solution that is better adapted to the way that the patient thinks and sees things.”*[10] In many cases, this helped participants make choices and take action.

4. Legitimizing the illness experience

Participants provided many examples in which a trust-based relationship combined with a good understanding of their situation contributed to the family physician’s comprehension of the various feelings (anger, powerlessness, grief…) they experienced in regard to their health. For seven participants who had no diagnosis or showed important distress, recognizing, naming and legitimizing these feelings were necessary steps towards a greater acceptance of the situation and the development of adaptation mechanisms.

A woman who had been suffering for two years from an undiagnosed disease told us: *“They are doctors who want to help me… because for me, not knowing what it is, is difficult”*[21]*.*

5. Acknowledging and promoting the patient’s expertise

As explained by the participants, knowing their situation, developing a good relationship, and demonstrating professional competence increased the physician’s credibility in their eyes. Participants told us that this could foster openness to discussing certain changes, awareness and ability to take action.

*“He always had the right way to make me understand things that I really did not want to understand”*[21]*.*

Eighteen participants explained that their family physician was in a good position to help them become aware of their strengths and develop self-confidence and expertise in regard to healthy habits and self-care.

*“Because he… gives me what it takes to continue”*[4]*.*

6. Helping the patient maintain hope

This sense of trust has also often placed the family physician in a privileged position to encourage the patient in maintaining realistic hope during difficult moments. Eleven participants addressed this notion of hope.

*“I am leaving here [physician’s office] like full of life to start again”*[10]*.*

## Discussion

In-depth interviews with patients affected by chronic diseases were carried out to identify family physician enabling attitudes and behaviours to promote patient empowerment. To this end, participants expressed that the family physician could foster their empowerment by 1) developing a partnership with them; 2) promoting their interests in the health care system; 3) knowing and starting from their personal situation; 4) legitimizing their illness experience; 5) acknowledging their strengths and promoting their expertise and 6) helping them maintain hope.

Participants clearly stressed the importance of a partnership with their family physician, a trust-based relationship being an important part of this alliance. Even if many authors have already stressed the importance of this relationship for patients [27-30], our study goes further by suggesting a link between this relationship and patient empowerment. Another recent qualitative study explored general practitioner and patient experiences of managing chronic illness in primary care with a particular focus on holding relationships [30]. Both physicians and patients emphasized the importance of pre-existing knowledge of past life-story, and valued holding as a potential tool for changing health-related behaviours.

However, a partnership did not mean that all participants in our study necessarily wanted to play an equivalent role in decision-making with their physician. They desired different levels of involvement in respect to certain personal characteristics, the nature of their chronic diseases and their relationship with their physician, as already described in the literature [31]. The desire to be involved in decision-making would be highly heterogeneous, thus an individualized approach for each patient may be needed [32].

Participants reported that difficulties in accessing a saturated care system could play a detrimental role in their process of empowerment. For most of them, another important issue was the involvement of their family physician in facilitating accessibility and improving continuity and coordination of care. This patient perception seems in conflict with that expressed by many physicians who consider accessibility to be beyond their control [33]. Other studies have also documented that organisational aspects such as waiting time, courtesy of office staff, being able to communicate with the physician and access to physicians would have impact on doctor-patient relationship [12,13,34]. Physicians should be aware of this impact. The potential gap between patient and physician perceptions regarding the physician’s role in organizational aspects of care deserves further evaluation.

In chronic disease management, some studies documented the importance of knowing and starting from the patient’s personal situation and adapting medical approaches to their expectations, desires, concerns and lifestyles [10,12,35,36]. In the enablement process, a good understanding of the patient’s situation would allow physicians to maximize the impacts of their interventions, helping patients develop empowerment in making choices and taking action.

The few studies on patient perception reporting the importance of recognizing, naming and legitimizing the illness experience, mainly involved patients with important distress related to conditions such as fibromyalgia [12,37] or cancer [35]. In our study, participants who had no clear diagnosis also needed acknowledgement of their condition to enhance their empowerment. In these circumstances, having the possibility to express feelings associated with the absence of a diagnosis was required to develop adaptation mechanisms.

Recognition and promotion of patient expertise were already described as key elements to promoting patient empowerment [3,38,39]. A secondary analysis of qualitative data sets from two studies with patients suffering from different chronic diseases — Type I Diabetes or Environmental Sensitivities — also demonstrated the necessity for physicians and nurses to value patient competence [40]. Participants in our study clearly stressed this important issue.

Many qualitative studies with patients and their family had shown the importance of offering realistic hope in palliative or end-of-life care [41-45]. Our study documented that patients with chronic disease need their family physician to provide hope, even if their lives are not compromised.

One limitation of our study is that the perception of people living in extreme poverty was scarcely explored. In addition, physicians involved in our study may have primarily called upon patients with whom they had a good relationship. Nonetheless, we asked participants to discuss previous encounters with other family physicians, which allowed us to ask questions about different relational experiences. In fact, many talked about difficulties experienced with other physicians and the impact on their empowerment. Further research is required prior to transferring these results to culturally different and older populations. Participants in our study had the same family physician for at least one year. The results are not transferable to situations where people with chronic conditions have no primary care physician and receive care from specialists only. Finally, although patient perceptions was a good starting point to identify enabling attitudes and behaviours, physician perceptions should also be solicited in further studies.

## Conclusion

According to patients, family physicians should try to form effective partnerships with them to promote their empowerment. Physicians’ role in furthering accessibility, continuity and coordination of care should be recognized. A trust-based relationship and a good understanding of the patient’s situation combined with professional competence increases physicians’ credibility in their eyes. This credibility could foster patient openness to discussing certain changes, and increasing awareness and ability to take action. The patient’s confidence in his or her family physician also places the health professional in a privileged position to encourage the patient maintain realistic hope.

## Competing interests

The authors declare that they have no competing interests.

## Authors’ contributions

CH, DST, GB and WH conceived and designed the study. CH, ML and MEP collected the data. All authors participated in the data analysis. CH and ML drafted the manuscript. All authors revised and approved the final manuscript.

## Support

This study received financial support from the CIHR Applied Research Chair – Health Services and Policy Research on Chronic Diseases in Primary Care (Canadian Institutes of Health Research-Institute of Health Services and Policy Research, Canadian Health Services Research Foundation and Centre de santé et de services sociaux de Chicoutimi).

## Pre-publication history

The pre-publication history for this paper can be accessed here:

http://www.biomedcentral.com/1471-2296/14/8/prepub

## Supplementary Material

Additional file 1 **Appendix 1. **Interview guide Click here for file

Additional file 2 **Appendix 2. **Characteristics of each study participant Click here for file
