# Supplementary material for: Family physician enabling attitudes: a qualitative study of patient perceptions
Source: BMC Fam Pract. 2013 Jan 10;14:8. doi: 10.1186/1471-2296-14-8 (PMC3556105; doi:10.1186/1471-2296-14-8)
Supplement: Additional file 1 — Appendix 1. Interview guide [file 1471-2296-14-8-S1.docx]

**Appendix 1 Interview guide**

INSTRUCTIONS FOR THE INTERVIEWER

- Read the consent form out loud to the patient. Provide additional explanations when necessary. Regularly check if the patient has questions and understands the content of the form.
- In light of this information, if the participant agrees to participate, have him/her sign two copies of the form. Leave one with the participant, the other is to be kept in the designated secured place.
- **Remind the person that the objective of the research is to better comprehend which attitudes of the family physician (words, ways of doing, ways of being, actions) can foster the autonomy of the patient living with chronic conditions (skill development, self-care, behaviour change, management of feelings in regard to the chronic disease...).**
- Specify to the patient that s/he may terminate the audio-recording at any time, that s/he can ask that certain excerpts be erased without any prejudice.
- Explain to the patient that the interview content is confidential and mention the main measures that will be taken to preserve his/her anonymity.
- **Specify that we are interested in his/her point of view, his/her perceptions, his/her experience. In that sense, there are no right or wrong answers to the questions.**
- **Briefly explain the sequence of the interview:**

**“You have been meeting with your family physician for a certain number of years and for certain health issues. During this interview, I would like you to tell me about the encounters you’ve had with your physician.”**

- Remind the person that there is a sociodemographic questionnaire to complete at the end of the interview.

Inspired and adapted from guides developed by St-Cyr Tribble et al. (2003 and 2007) and Godbout (2007) that have already been validated for their content.

Question #1

**Before beginning the first part of the interviewer, I would like you to provide the following information:**

1. **Since when have you been meeting your family physician?**
2. **What is the frequency of your encounters?**
3. **How are your encounters planned?**
4. **What is the average length of one encounter?**
5. **Have you seen other family physicians before?**

Interview Part 1: Your experience with your health situation

Question #2

**Tell me about your health status.**

Themes to explore if not addressed spontaneously:

- Has s/he had one or many chronic diseases? Which one(s)?
- Since when?
- What has been the general evolution of the disease(s)?

Question #3

**Do you take any medication?**

**Is there any specific monitoring to do? Tell me more about it... (Diet, physical activity, measuring blood pressure, blood glucose, quit smoking...) or pain management?**

Themes to explore if not addressed spontaneously:

- Patient involvement
- Difficulties
- Strengths, skills

Question #4

**Tell me about the repercussions of your health status on your life.**

The interviewer can use the conditions mentioned in answer to question #2 to stimulate the discussion.

Themes to explore if not addressed spontaneously:

- How did s/he feel at the beginning (despair, powerlessness, little feeling of control, awareness, motivation, ...)
- How does s/he feel now?
- Changes brought on by this situation (grief, behaviour change, self-care...)
- What the patient went through or is going through in terms of needs, difficulties, preoccupations, problems.
- His/her perception of his/her strengths, skills, limits

**In general, how would you describe your self-confidence?**

Interview Part 2: Your encounters with your family physician

The second part of the interview concerns your meetings with your family physician.

Question #5

**How do you feel during your encounters with your family physician? Explain.**

Themes to explore if not addressed spontaneously:

- Have the patient specify his/her feelings and how they have evolved over time.
- What brings the patient to feel this way

Question #6

**Are there things that have left a positive mark on you in your encounters with your family physician? If yes, tell me about them.**

Themes to explore if not addressed spontaneously (if the patient says no, enumerate these themes; if the patient responds yes, name those that were not addressed):

- Words
- Ways of doing
- Ways of being (attitudes)

**Are there things that have left a negative mark on you in your encounters with your family physician? If yes: tell me about them.**

**Are there things that have left a negative or positive mark on you in your encounters with other family physicians? If yes: tell me about them.**

Question #7

**Among all of your family physician’s attitudes, which one do you prefer?**

Question #8

**If you had suggestions to make to your physician about your encounters, what would they be?**

Make up scenarios if needed...

Question #9

*Synthesize the important elements of the context that was addressed up until now to introduce question #9 by personalising the question...*

**Give me an example of a situation in which your family physician helped you become more autonomous in your experience with your health status** (or with disease x).

The interviewer explains that it can be one or many of the following results: 1) becoming aware of one’s strengths, of one’s needs; 2) developing self-esteem; 3) decrease in anxiety or sadness; 4) improved decision-making; 5) learning of skills; 6) taking action (changing things).

Question #10 (if it’s relevant to the answers to the question asked in #9)

**Can this help you in other spheres of your life? In what way?**

Themes to explore if not addressed spontaneously:

- Social activities
- Family relations
- Well-being
- ...

Question #11

**Tell me, on a scale from 1 to 10, if you have the impression that your encounters with your family physician help you become more autonomous in living with your health situation.**

**Can you elaborate on this score?**

Question #12

**Are there other aspects that we did not address during this interview and that you would like to share with me?**

Themes to explore if not addressed spontaneously:

- Impression that the collaboration is helpful or not
- Questions that were embarrassing or not, difficult to understand or not, difficult to answer or not.

**References**

St-Cyr Tribble, D., Gallagher, F., Bell, L., Caron, C., Godbout, P., LeBlanc, J., Morin, P., Xhignesse, MA. (2006-2009). Intervention d’Empowerment, point de vue de l’intervenant et de la clientèle des soins à domicile. Institut de Recherche en Santé du Canada (IRSC).

**Recent publications**

Godbout, P., St-Cyr Tribble, D. (2009). L’apport de l’observation in situ pour décrire les interventions infirmières d’habilitation à l’autoprise en charge de sa santé dans le secteur des soins à domicile. Moncton : Presses de l’Université de Moncton.

St-Cyr Tribble, D., Gallagher, F., Bell, L., Caron, C., Godbout, P., LeBlanc, J., Morin, P., Xhignesse, MA. Voyer, L., Couture, M. (2008). Empowerment interventions, knowledge translation and exchanges: perspectives of home care professionals, clients and caregivers. Health Services Research: BioMed Central Edition, www.biomedcentral.com/qc/1472-6963/8/177

St-Cyr Tribble, D., Gallagher, F., Couture, M., Voyer, L., Bell, L., Caron, C., Godbout, P., LeBlanc, J., Xhignesse, MA. Carignan, H. (2007-2009). Élaboration des guides d’entrevue et d’analyse de contenus de clients, d’intervenants et de proches-aidants : Groupe de recherche sur l’intervention d’Empowerment en première ligne, Université de Sherbrooke, document inédit.

Godbout, P. (2007). Habilitation à l’autoprise en charge de sa santé, représentations des infirmières des soins à domicile. Thèse de doctorat en sciences cliniques (sciences infirmières). Sherbrooke : Université de Sherbrooke.

St-Cyr Tribble, D., Paul, D., Gallagher, F., Archambault, J. (2003). Les pratiques d’Empowerment en première ligne: compréhension et évaluation de l’efficacité des interventions infirmières et psychosociales conduites auprès de parents. Rapport de recherche, Série B, Études scientifiques. Sherbrooke : Centre d’innovation, de recherche et d’enseignement, CLSC de Sherbrooke, 2e édition.
