# Supplementary material for: Family physician enabling attitudes: a qualitative study of patient perceptions
Source: BMC Fam Pract. 2013 Jan 10;14:8. doi: 10.1186/1471-2296-14-8 (PMC3556105; doi:10.1186/1471-2296-14-8)
Supplement: Additional file 2 — Appendix 2. Characteristics of each study participant [file 1471-2296-14-8-S2.docx]

**Appendix 2 Characteristics of each study participant**

| Participant | Sex and age | Education | Revenue (CAN$) | Marital status | Self-reported chronic diseases |
| --- | --- | --- | --- | --- | --- |
| 1 | H56 | Grade 8 to 12 | 50,000 or more | Married / common law | Hyperlipidemia |
| 2 | F63 | University | 10,000 to 19,999 | Married / common law | Stroke, depression antecedents, asthma, hyperlipidemia |
| 3 | F47 | College | 20,000 to 29,999 | Separated or divorced | Fibromyalgia, hypertension |
| 4 | F60 | Grade 8 to 12 | 10,000 to 19,999 | Single | Osteoarthritis, hyperlipidemia |
| 5 | H65 | University | 40,000 to 49,999 | Married / common law | Osteoarthritis, diabetes, hyperlipidemia, hypertension, heart disease |
| 6 | F62 | Grade 8 to 12 | Less than 10,000 | Separated or divorced | Osteoarthritis, diabetes, hyperlipidemia, hypertension |
| 7 | F54 | College | Less than 10,000 | Single | Depression antecedents, osteoarthritis, heart disease |
| 8 | H62 | College | 50,000 or more | Married / common law | Asbestosis, depression antecedents, hyperlipidemia |
| 9 | F53 | College | 50,000 or more | Married / common law | Depression antecedents, osteoarthritis, chronic bronchitis, diabetes, hyperlipidemia, hypertension, heart disease |
| 10 | F75 | College | 50,000 or more | Married / common law | Arthritis, diabetes, hyperlipidemia, hypertension, heart disease |
| 11 | F48 | College | 50,000 or more | Married / common law | Depression antecedents, diabetes |
| 12 | H64 | College | 40,000 to 49,999 | Married / common law | Depression antecedents, hyperlipidemia, hypertension |
| 13 | H54 | University | 50,000 or more | Single | Diabetes, hyperlipidemia, hypertension |
| 14 | F63 | Grade 8 to 12 | 40,000 to 49,999 | Widowed | Depression antecedents diabetes, hyperlipidemia, hypertension |
| 15 | F64 | University | 40,000 to 49,999 | Separated or divorced | Heart disease |
| 16 | F47 | University | 50,000 or more | Married / common law | Diabetes, hyperlipidemia, hypertension |
| 17 | H55 | University | 50,000 or more | Married / common law | Depression antecedents, hyperlipidemia, heart disease |
| 18 | H74 | College | 30,000 to 39,999 | Separated or divorced | Depression antecedents, hyperlipidemia, heart disease |
| 19 | H69 | University | 50,000 or more | Married / common law | Diabetes, hypertension |
| 20 | H72 | Grade 8 to 12 | 20,000 to 29,999 | Married / common law | Hyperlipidemia |
| 21 | H61 | Grade 1 to 7 | 10,000 to 19,999 | Married / common law | Arthritis, diabetes, hyperlipidemia, hypertension, heart disease |
| 22 | F67 | University | 50,000 or more | Married / common law | Asthma, hyperlipidemia, hypertension |
| 23 | F70 | Grade 8 to 12 | 10,000 to 19,999 | Married / common law | Osteoarthritis, asthma |
| 24 | H64 | Grade 8 to 12 | 50,000 or more | Separated or divorced | Depression antecedents, hyperlipidemia, hypertension, heart disease |
| 25 | F66 | Grade 8 to 12 | 40,000 to 49,999 | Married / common law | Osteoarthritis, diabetes, hypertension |
| 26 | F56 | Grade 8 to 12 | 40,000 to 49,999 | Married / common law | Anxiety, hypertension, osteoporosis |
| 27 | F35 | University | 10,000 to 19,999 | Married / common law | Anxiety |
| 28 | H62 | Grade 8 to 12 | 10,000 to 19,999 | Single | Stroke, depression antecedents, hyperlipidemia, hypertension |
| 29 | H55 | University | 50,000 or more | Married / common law | Heart disease |
| 30 | F69 | Grade 8 to 12 | 30,000 39,999 | Widowed | Depression antecedents, osteoarthritis, heart disease |
